# What drives a normal relation between T-CD4 and T-CD8?

**DOI:** 10.1186/1758-2652-13-S4-P152

**Published:** 2010-11-08

**Authors:** R Badura, RJG Antunes, N Janeiro, C Afonso, F Antunes

**Affiliations:** 1 Hospital Universitário de Santa Maria, Dept for Infectious Diseases, Lisbon, Portugal; 2 Faculdade de Medicina, University of Lisbon, Lisbon, Portugal; 3 Hospital Universitário de Santa Maria and Faculdade de Medicina, University of Lisbon, Dept for Infectious Diseases, Lisbon, Portugal

## Introduction

Inversion of the CD4/CD8 ratio, in the context of HIV infection, is a frequent finding, even though, if the patient has been on prolonged antiretroviral therapy. Nonetheless, in a small proportion undergoing antiretroviral therapy, this relation normalizes.

## Purpose of the study

With the aim to investigate frequency and the population characteristics of this occurrence we proposed a retrospective analysis in our outpatient HIV clinic in Lisbon.

## Methods

Patients with a proven inversion of CD4/CD8 ratio before the beginning of antiretroviral treatment and who, in the last five years, re-inverted to a normalized ratio (above one) in at least two consecutive estimations were eligible. Variables analyzed included: gender, age, former opportunistic infections, CD4+ nadir, viral load, length of antiretroviral therapy and therapeutic regime. Obtained data was tested for correlation and statistical significance using student T-test.

## Results

Of 1.750 patients on antiretroviral therapy, 119 patients reverted to a normal T-CD4/CD8 ratio in the last five years. Six of these where infected with HIV-2, five did not maintain reversion, which lead to a 108 patients with a true reversion, corresponding to 6% of the population. Not being able to access the files in 18 cases, the analysis is based on 90 patients. The mean-time of antiretroviral therapy before reversion of the CD4/CD8 ratio was 69 month. The distribution reveals two peaks: one around month 40th and another around month 130th, probably related to former therapeutic regimes. No significant correlation was found if time-of-antiretroviral-therapy-until-reversion was analysed with respect to impact by opportunistic infection, T-CD4 nadir, viral load, gender or the ability of the antiretroviral therapy to penetrate the CNS. Though, not a frequent finding, it seems to be constant and strongly correlated to time-of-antiretroviral-therapy with a correlation of Pearson of 0,501 (p=0.01).

## Conclusions

These findings suggest that the CD4/CD8 might be a marker for the follow-up in patient undergoing antiretroviral therapy. Its real impact, though, needs further investigation especially with respect to T-CD8 specific cytotoxicity and activation.

